# Effect of Auditory Distraction on Working Memory, Attention Switching, and Listening Comprehension

**DOI:** 10.3390/audiolres11020021

**Published:** 2021-05-28

**Authors:** Naveen K. Nagaraj

**Affiliations:** Cognitive Hearing Science Lab, Communicative Disorders & Deaf Education, Utah State University, Logan, UT 84322, USA; naveen@usu.edu

**Keywords:** working memory, attention switching, listening in noise

## Abstract

The effect of non-informational speech spectrum noise as a distractor on cognitive and listening comprehension ability was examined in fifty-three young, normal hearing adults. Time-controlled tasks were used to measure auditory working memory (WM) capacity and attention switching (AS) ability. Listening comprehension was measured using a lecture, interview, and spoken narratives test. Noise level was individually set to achieve at least 90% or higher speech intelligibility. Participants’ listening comprehension in the presence of distracting noise was better on inference questions compared to listening in quiet. Their speed of information processing was also significantly faster in WM and AS tasks in noise. These results were consistent with the view that noise may enhance arousal levels leading to faster information processing during cognitive tasks. Whereas the speed of AS was faster in noise, this rapid switching of attention resulted in more errors in updating items. Participants who processed information faster in noise and did so accurately, more effectively switched their attention to refresh/rehearse recall items within WM. More efficient processing deployed in the presence of noise appeared to have led to improvements in WM performance and making inferences in a listening comprehension task. Additional research is required to examine these findings using background noise that can cause informational masking.

## 1. Introduction

In real-life, we perform a variety of cognitive tasks such as, reasoning, writing, reading, and listening in the presence of interfering background noise. Considerable evidence supports that background noise/sounds have a detrimental effect on cognitive task performance, such as verbal short-term memory and reading comprehension [1,2,3,4]. Often this effect of noise or sound is discussed in terms of cross-modal distraction, called irrelevant sound effect (ISE). Serial recall is one of the most commonly used tasks to study the ISE and findings from a vast body of literature suggest that serial recall of visually presented stimuli is impaired in the presence of sound, irrespective of whether the sound is presented during the presentation of “to be remembered” stimuli, or during the retention interval before recall [5,6,7,8]. One critical factor that determines the magnitude of ISE is whether irrelevant sound is static or changing. Thus, a sequence of changing sounds such as “ba, da, ba, ka, ba, ta,…” produces greater disruption to serial memory recall compared to a sound sequence that does not change “ba, ba, ba, ba, ba, ba,…”. Jones and colleagues proposed the “changing state hypothesis” to account for ISE [1]. According to this hypothesis, change in the irrelevant sound is the key feature that disrupts serial recall. Changing state effect is mainly seen if the task requires recall of items in strict serial order, but not when asked to recall freely in any order [1,9,10,11]. The meaning of irrelevant sound has been found to have no effect on serial recall performance [1,12,13,14]. However, semantic information in irrelevant sound can significantly disrupt language-based tasks, such as reading comprehension [3,4,15], reasoning [16], and writing [17]. This effect of ISE is also known as semantic interference or informational masking in the speech perception literature. Changing state effect and semantic interference is thought to reflect auditory distraction caused by interference-by-process. According to this view, auditory distraction arises due to a conflict between contemporaneous processes; one that is applied automatically to the irrelevant sound stream and a deliberate process applied to the focal task material [2,11]. Several findings suggest that low level of noise may enhance arousal levels, leading to faster and better hearing performance [18,19,20].

The magnitude of the changing state effect, however, is not found to be associated with working memory capacity (WMC) [21,22,23,24]. In contrast to the changing state effect, several studies have found that WMC is related to individuals’ susceptibility to semantic interference [21,25,26]. Individual differences in WMC also predict a variety of complex cognitive functions, such as language comprehension, reasoning, fluid intelligence, and problem solving [27,28,29,30,31,32]. This high degree of association between complex cognitive tasks and WMC is believed to be in part due to the domain-general executive attention mechanism that is used to overcome distraction or interference in a wide range of tasks [30]. The executive attention system is also responsible for controlling and organizing how incoming information is processed within the working memory (WM) system [33,34,35,36]. Attention control is a fundamental cognitive skill that is crucial for listening as it occurs in real-life settings where multiple irrelevant stimuli compete for attention. WMC is thought to reflect individuals’ ability to maintain attention control over task relevant information while filtering out task irrelevant information [34,36,37,38,39]. However, little is known about WM capabilities, especially executive processing that may underpin listening against a background of noise. One executive process that may play a role in offsetting the loss of information from the WM system is attention control; the system switches attention between processing incoming information, while maintaining extant information within WM [40,41,42,43,44,45].

Understanding spoken discourse requires successful operations at different levels of linguistic analysis, such as phonological, lexical, syntactic, and semantic processing of incoming speech. Contextual cues such, as lexical, syntactic, prosodic, and semantic information stored in long-term memory, also play a significant role in speech understanding [46,47]. To successfully understand speech in noise, these linguistic analyses must be carried out with the proper allocation of cognitive resources, such as WM and attention [48,49,50,51,52]. The fact that all such processing is achieved in real-time makes listening comprehension in noise dynamic and complex. Working memory has been found to be crucial for performing complex tasks such as listening and reading comprehension because the intermediate products of linguistic processing have to be kept active until the listener/reader is able to understand the message in its entirety [29,53]. The attention-mediated portion of the executive mechanism within the WM system is responsible for active processing of new speech information and prevention of the decay of information. It is also believed that individuals alternate quickly between processing and storage during complex cognitive tasks such as reading or listening [40,41,42,43,44,45]. When processing is unnecessary, attention is switched to maintenance mode to refresh memory before returning to process new information. The executive attention system is responsible for controlling and organizing how the incoming information is processed within the WM system [33,34,35,36]. Since WMC is limited, resources must be shared between maintenance and processing. As the difficulty of the listening situation increases due to noise, resources available for processing and maintenance are heavily taxed, thus increasing the odds of forgetting or errors in speech understanding. Attention is also required to inhibit distracting stimuli such as noise and to selectively attend to speech for better understanding of spoken messages embedded in noise [34,54,55,56]. Many everyday listening situations require focusing of attention to relevant information, inhibition of irrelevant information, access, and retrieval of information from long-term memory and experience.

Given the importance of attention control to WM and listening in noise, the present study was designed to investigate the effect of irrelevant fluctuating noise on listening comprehension, attention switching (AS), and WM. Most studies in the literature have studied the ISE using cross-modal tasks in which the effect of auditory sounds was examined on visual serial recall. In this study, listening-based cognitive tasks were used because it was assumed that noise would have greater interference on auditory than visual-based tasks. The aim was to understand whether attentional switching within the WM is modulated by the presence of irrelevant fluctuating noise and whether the effectiveness of attention switching in noise was associated with WM performance. Furthermore, a second aim was to evaluate the effect of noise on listening comprehension. A natural listening task consisting of lectures, interviews, and spoken narratives was used to measure listeners’ speech understanding ability in noise. Cognitive tasks were administered in quiet and noise utilizing a fixed time window for processing and response. Control of time is not only essential to tax the central executive attention during WM tasks [42,43,57,58], but also to keep the task consistent between the quiet and noise conditions for comparison. Fluctuating, non-intelligible speech-like noise was used without semantic interference or informational masking. However, the intensity of noise was controlled in such a way as to achieve at least 90% or higher speech intelligibility for stimuli used in the cognitive tasks. These were essential to ensure that performance differences in cognitive tasks in the presence of noise were not because of masking (reduced intelligibility or informational masking), but due to its interference as a result of changing state.

## 2. Materials and Methods

### 2.1. Participants

Fifty-three adults with normal hearing between 18–37 years (*M* = 22.5 years) took part in this study. All participants were native monolingual speakers of American English and had hearing within normal limits as determined by hearing screening. Participants were also screened to rule out cognitive impairment using the “Six-item cognitive screener” [59]. The study was conducted in full compliance with Ohio University, Institution Review Board procedures.

### 2.2. Stimuli and General Procedure

Within-subject repeated measures design was used. All participants performed the tasks in the same order to avoid individual variability due to task order [60]. However, the noise and quiet conditions were counterbalanced to minimize order effects. Once a participant was eligible to take part in the study, two sessions of about two hours each were required to complete all the tasks. Digits one through nine excluding seven (because it was bi-syllabic) were used for the forward and backward digit span task. Monosyllabic nouns and verbs taken from CNC and NU-6 [61,62] word list were used for the AS task. Digits and CNC monosyllabic words were used for the auditory WM task. Digits and monosyllabic words in isolation were recorded by a female native English speaker in a sound attenuating booth using a Sennheiser microphone (Sennheiser 845 S) and Avid pro tools pre-amplifier (Mbox Pro). All stimuli were edited to normalize duration and level, using Adobe Audition 3.0 and Avid Audio Protools 8.1. The duration of each digit and the monosyllabic words were compressed or lengthened to make them equal to 500 ms and amplitude was normalized to have equal RMS value. Finally, visual inspection using spectrogram and waveform analysis along with listening check was performed for all the stimuli by four adult listeners independently, to ensure that there was no audible distortion. Participants were tested individually in a double-walled sound-treated room. All tasks were created and delivered using E-Prime 2 software (Psychology Software Tools, Inc. PA, USA) on a PC with sound card (Creative Labs Soundblaster X-Fi). Stimuli were played out from the PC at a sampling rate of 22,000 Hz and were fed to Tucker-Davis Technologies (TDT) PA5 programmable attenuator and power amplifier (Crown CTs-4200) before being sent to the loud speaker (B&W DM601 S3). All participants listened to the stimuli binaurally presented through a loudspeaker, located at 0^0^ azimuth and at a distance of one meter from the participants’ head. The level of speech stimuli was fixed at 65 dB SPL. This level was selected because it represents the average conversational level and should elicit highest speech intelligibility. The International Collegium for Rehabilitative Audiology (ICRA) noise [63] was used as a competing noise for all the tasks, since it has the same spectral and temporal characteristics as that of multi-talker noise spoken at a normal vocal effort (Track 7 from ICRA CD).

Multi-talker babble is the most common environmental noise encountered in everyday situations and babble affects speech perception more than any other type of noise [64]. ICRA was selected over multi-talker babble for this study, mainly because it lacks informational masking ability while being similar to multi-talker babble [63]. Noise in the present study was used as a distractor to increase the cognitive load while performing cognitive and speech tests. Informational masking noise, such as a multi-talker babble, was not selected to avoid any further increase in masking and listening effort while performing the experimental tasks. For noise conditions the intensity of the speech stimuli was kept constant at 65 dB SPL and the intensity of ICRA noise was varied to obtain the desired speech to noise ratio (SNR) using TDT PA 5 programmable attenuator. All stimuli were calibrated using Brüel and Kjær half-inch free-field microphone (4189) with hand-held sound level meter (Type 2250).

### 2.3. Speech Intelligibility Tests to Determine SNR for 90% Intelligibility

*Stimuli.* Standardized clinical word- and sentence-in-noise tests were used to measure speech intelligibility and to adjust the noise level for cognitive and listening comprehension tasks for each individual based on these scores. Open-set Maryland CNC monosyllabic words [61] and Quick SIN test sentences [65] were used to measure word and sentence recognition abilities in quiet and noise.

*Procedure.* Word and sentence recognition was measured in quiet and five levels of noise by randomly varying the signal-to-noise ratio (SNR) from +10 to −10 dB SNR in 5 dB steps to obtain a psychometric function. Each list in the word recognition task consisted of 50 monosyllabic words. Each list in the sentence recognition task consisted of twelve sentences with a total of 60 keywords. Participants were tested in both quiet and five fixed noise conditions using separate lists. All the words were presented with the carrier phrase “Say the ____ again” with 3.5 s inter-stimulus interval. Participants were asked to repeat what they heard during that time. Sentences were presented in 5 s inter-stimulus intervals and participants were asked to repeat the sentence they heard during that time. The presentation order of the stimuli and the SNR condition was randomly assigned to each participant to minimize order effects. The numbers of words correctly recalled were scored. The noise level required to achieve 90% correct word and sentence recognition was obtained for each participant using three parameter sigmoidal curve fitting [66].

### 2.4. Attention and Memory Tasks

All the attention and memory tasks were administered in quiet and noise conditions. The intensity of noise was adjusted based on individuals’ SNR for 90%-word recognition scores.

### 2.5. Auditory Attention Switching

This task was designed to measure listeners’ ability to switch the focus of attention from one mental set to another and is based on paradigms used in previous studies [36,67].

*Stimuli.* Ten nouns (girl, hen, lawn, door, cat, coke, shirt, lake, rug, pearl) and ten verbs (ran, reap, get, said, learn, beg, thank, sing, join, give) were selected from standardized CNC and NU-6 monosyllabic word lists based on a survey. To verify that there was no ambiguity between nouns and verbs selected, an online survey was conducted. In this survey participants were given a random list of 128 words and were asked to determine whether a word was a “Noun” or “Verb” or “Both Noun and Verb” or “Not Sure”. A total of 27 native English-speaking adults completed the survey. The results for each word were tallied. Only those words that received greater than 96% on classification as either “Noun” or “Verb” were used for this task.

*Procedure.* Each participant listened to a randomized list of 10 nouns and 10 verbs. For any given presentation, participants were instructed to judge the category to which the word belonged, i.e., “Noun” or “Verb” and keep track of the number of nouns and verbs. Subsequent presentation of stimuli was controlled by the subject using two-interval forced choice response on the response box. The time from the beginning of noun or verb (word) presentation and the button press was recorded as the reaction time. The time-window to respond was fixed at 2000 ms based on pilot data. Participants were asked to respond as fast as possible immediately after the word presentation ended. At the end of a set, they were cued to verbally recall the total number of Nouns and Verbs in any order they preferred. The number of words in each set was randomly varied from 12 to 16. Words were not repeated within a set. Each set consisted of switch and non-switch presentations and there was a total of 40 sets. A presentation was labeled as a “switch” presentation if a given word was from a different category than the previous word (e.g., “hen” being in the “noun” category followed by “beg” being in the “verb” category or vice-versa). If two consecutive words were from the same category (e.g., “girl” followed by “lake” both being in the “noun” category), then the presentation was labeled as a “nonswitch” presentation. Half of the sets were classified as “High-Switch” and the other half as “Low-Switch”. “Low-Switch” sets consisted of only 25% category switches between the noun and verb categories and the “High-Switch” sets had 50% or more category switches between noun and verb. The outcomes measured from this task were (a) overall accuracy of verbal recall of noun and verb counts (AS updating accuracy), (b) response time indexing speed of AS from one category to the other (switch RT), and (c) response time indexing speed of AS within each category (nonswitch RT).

### 2.6. Auditory Working Memory

An auditory-based WM task was designed to combine both storage and processing aspects in one task under the assumption that both storage and processing rely on the same attention resource [28,55,68]. The general procedure of the auditory WM task was similar to the forward digit span task, but included a processing part after each digit presentation. In this complex WM task, listeners were asked to remember a sequence of digits presented (storage part of the task) along with a processing task. The processing part of the task required participants to hear four words and make a three-alternative forced choice. Participants were required to decide if “word 1 = word 3” (choice 1), “word 2 = word 4” (choice 2), “word 1 = word 3 and word 2 = word 4” (choice 3). The inter-stimulus interval between the four words to be processed was kept constant to 500 ms. The processing part of this task was devised to measure participants’ word recognition and discrimination ability. Before the first processing episode of the block, and 100 ms after each subsequent response, a digit was presented to be remembered for later recall. 500 ms after the digit presentation, the next processing episode was presented. After each processing episode, the participants were required to press the corresponding button on the response box to indicate their response choice. Maximum time window to respond for the processing task was fixed at 2000 ms based on pilot data. Participants were instructed to respond as quickly as possible immediately after the processing stimuli presentation ended. The response window was fixed because evidence suggests that substantial delay between stimuli in a block may give participants more time to rehearse the digits to be remembered, which can make the task more like a STM task [57,69,70]. The number of digits and processing items in a given block varied from two to seven. Previous research on reading span tasks have shown that this range is adequate for reliably measuring WM span in college student populations [30,57]. The task consisted of a total of 18 total trials. Each block was repeated three times with different items. Each block was also randomly presented to reduce order effects.

The correct identification from the three-alternative choices in the processing task was recorded as processing accuracy. The time between the beginning of the fourth word on the processing task and the button press response was recorded as processing time. At the end of each block, participants had to recall the sequence of digits presented in correct serial order. Recall accuracy of digits was scored similar to the forward recall task to obtain a measure of WMC. The proportion scoring used in the current study has been found to be reliable [57] and correlates better with verbal SAT scores [71].

### 2.7. Listening Comprehension Test

The Lecture Interview and Spoken Narratives (LISN) test was used to measure listening comprehension [72,73]. The LISN test closely represents natural listening conditions and has moderate to high internal consistency and reliability across a wide age-range (20–89 years). The LISN test was performed in quiet and in noise. The noise level was adjusted based on an individual’s SNR 90% sentence recognition scores as described in above section “Speech Intelligibility Tests to determine SNR for 90% intelligibility”.

*Stimuli.* All passages were from the original LISN test. Six passages: two lectures, two interviews, and two spoken narratives, each lasting approximately three to five minutes were presented at conversational speech level in quiet and noise. LISN test passages have been recorded by three male and three female professional actors in a natural or conversational style and have been normalized for RMS to minimize level difference between recordings.

*Procedure.* Passages were presented over the loudspeaker at 65 dB SPL in a sound treated room. Listening comprehension was measured in both quiet and noise conditions. The SNR for the noise condition was adjusted individually for each participant based on 90% SNR for sentence recognition task. This was done to ensure that the intelligibility of the passages was not affected in the presence of noise. After each passage, the listener was asked to respond to comprehension questions. For each passage standardized questions were presented to assess various aspects of listening comprehension. *Information questions* assessed participants’ ability to recall specific pieces of information. *Integration questions* assessed the ability to combine two or more separate pieces of information from the passage. Finally, *inference questions* assessed participants’ ability to derive inferences from the passage. Two questions were selected for each category and the order of questions was randomly presented. After each passage, the comprehension question and the response choices were presented on the computer screen. Participants were instructed to select the most appropriate choice by pressing a button on the response box. The outcomes were percent correct scores under each question category.

## 3. Results

Descriptive statistics for all experimental tasks are shown in Table 1. Reaction time data for both AS and WM tasks were analyzed only for those trials with correct responses. Any reaction time that was less than 200 ms and farther than +/−3 SD from the mean was removed (<1.8% of data was removed) from the analysis.

### 3.1. Auditory Attention Switching Task

**Figure 1:**
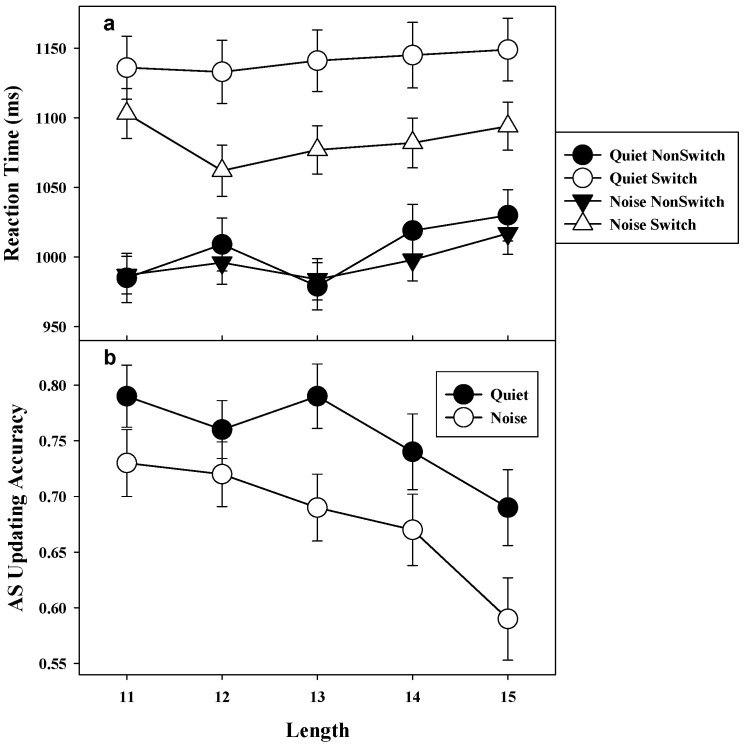
Mean switch and nonswitch reaction time by condition and set length (panel (**a**)). Mean attention switching updating accuracy by set length (panel (**b**)). Error bars depict one standard error of mean. AS = Attention Switching.

*Reaction time.* Analysis of the RT data for the AS task was done by a 2 (condition: quiet vs. noise) X 2 (switch: switch vs. nonswitch) X 2 (frequency: low vs. high) within-subject repeated measures analysis of variance (ANOVA). ANOVA revealed a main effect of condition, *F*(1, 52) = 4.7, *MSE* = 25,396, *p* < 0.05, *η*^2^ = 0.08, switch, *F*(1, 52) = 86.3, *MSE* = 15,046, *p* < 0.001, *η*^2^ = 0.62, and frequency, *F*(1, 52) = 140.7, *MSE* =2962, *p* < 0.001, *η*^2^ = 0.73. This suggested that responses on the AS task were slower in quiet (*M* = 1074.5 ms, *SD* = 213.9) than in noise condition (*M* = 1041.0 ms, *SD* = 163.2), responses for switch trials (*M* = 1113.1 ms, *SD* = 201.2) were slower than nonswitch trials (*M* = 1002.4 ms, *SD* = 162.2), and finally responses were slower for high frequency switch trials (*M* = 1089.1 ms, *SD* = 191.2) than low frequency switch trials (*M* = 1026.4 ms, *SD* = 185.5). The ANOVA also confirmed a significant condition by switch interaction, *F*(1, 52) = 18.4, *MSE* = 3385, *p* < 0.001, *η*^2^ = 0.27. Post-hoc analysis of condition by switch interaction was examined using Tukey–Kramer multiple comparison test. As shown in Figure 1a, results suggested that switch RT in quiet was slower than in noise condition (Mean Difference = 57.6 ms, 95% CI (36.4, 78.8)), but nonswitch RT was not different between the two conditions (Mean difference = 9.2 ms, 95% CI (−12.0, 30.4)). Furthermore, the cost of switching (difference between switch and nonswitch RT) in both quiet (Mean difference = 134.9 ms, 95% CI (113.7, 156.1)) and noise condition (Mean difference = 86.5 ms, 95% CI (65.2, 107.7)) was significantly different, indicating switch RT was longer than nonswitch RT in both conditions. Neither the condition by frequency interaction, nor the frequency by switch interaction was significant (*F* < 1). There was also no significant three-way interaction of condition, switch, and frequency *F*(1, 52) = 2.9, *MSE* = 805.8, *p* > 0.087, *η*^2^ = 0.05.

*Updating accuracy.* Accuracy for count updating for nouns and verbs in the AS task were subjected to 2 (condition: quiet vs. noise) X 2 (stimuli: noun vs. verb) X 2 (frequency: low vs. high) within-subject ANOVA. The omnibus test revealed a main effect of condition, *F*(1, 52) = 6.6, *MSE* = 0.09, *p* < 0.05, *η*^2^ = 0.11, stimuli *F*(1, 52) = 9.5, *MSE* = 0.007, *p* < 0.01, *η*^2^ = 0.15, and frequency, *F*(1, 52) = 33.3, *MSE* = 0.011, *p* < 0.001, *η*^2^ = 0.38. The result suggested that overall accuracy in quiet (*M* = 0.76, *SD* = 0.20) was higher than in noise condition (*M* = 0.68, *SD* = 0.21), as shown in Figure 1b. The results also suggested that frequency of switches negatively affected updating accuracy, with high frequency switch trials less accurate (*M* = 0.69, *SD* = 0.23) than low frequency switch trials (*M* = 0.75, *SD* = 0.21). The accuracy of counts for nouns (*M* = 0.73, *SD* = 0.22) were slightly better than the count for verbs (*M* = 0.71, *SD* = 0.23). Neither the condition by stimuli interaction nor the condition by frequency interaction was significant (*F* < 1). The three-way interaction comprising condition, stimuli, and frequency was also not significant (*F* = 0).

*Stimulus identification accuracy.* Accuracy of stimulus identification was subjected to 2 (condition: quiet vs. noise) X 2 (switch: switch vs. nonswitch) X 2 (frequency: low vs. high) within-subject repeated measures ANOVA. There was no main effect for condition, *F*(1, 52) = 0.39, *MSE* = 0.002, *p* > 0.5, *η*^2^ = 0.007, and frequency, *F*(1, 52) = 0.8, *MSE* = 0.0004, *p* > 0.37, *η*^2^ = 0.01 suggesting that identification accuracy was not significantly different between noise and quiet conditions and also between high and low frequency switch trials. The results revealed a main effect for switch, *F*(1, 52) = 41.1, *MSE* = 0.0006, *p* < 0.001, *η*^2^ = 0.44, indicating that stimulus identification accuracy was marginally less on switch trials (*M* = 0.96, *SD* = 0.04) than on nonswitch trials (*M* = 0.98, *SD* = 0.03). The only significant interaction was switch by frequency, *F*(1, 52) = 14.0, *MSE* = 0.0004, *p* < 0.001, *η*^2^ = 0.21.

### 3.2. Auditory Working Memory Task

*Recall accuracy (WM capacity)***.** Accuracy of digit recall on the WM task was subjected to 2 (condition: quiet vs. noise) X 6 (block length: 2, 3, 4, 5, 6, 7) repeated measures ANOVA. Main effect was significant for condition, *F*(1, 52) = 4.7, *MSE* = 0.077, *p* < 0.05, *η*^2^ = 0.08, indicating that the recall accuracy was significantly better in noise (*M* = 0.73, *SD* = 0.16) than in quiet condition (*M* = 0.68, *SD* = 0.16). There was a significant main effect of block length, *F*(5, 260) = 86.0, *MSE* = 0.029, *p* < 0.001, *η*^2^ = 0.62, and condition by block length interaction, *F*(5, 260) = 5.3, *MSE* = 0.029, *p* < 0.001, *η*^2^ = 0.09. Correlational analysis revealed a significant negative correlation between accuracy and block length for both quiet (r = −0.22, 95% CI (−0.32, −0.12), *p* < 0.01) and noise condition (r = −0.19, 95% CI (−0.29, −0.07), *p* < 0.01), which suggested that accuracy decreased with increase in number of items.

**Figure 2:**
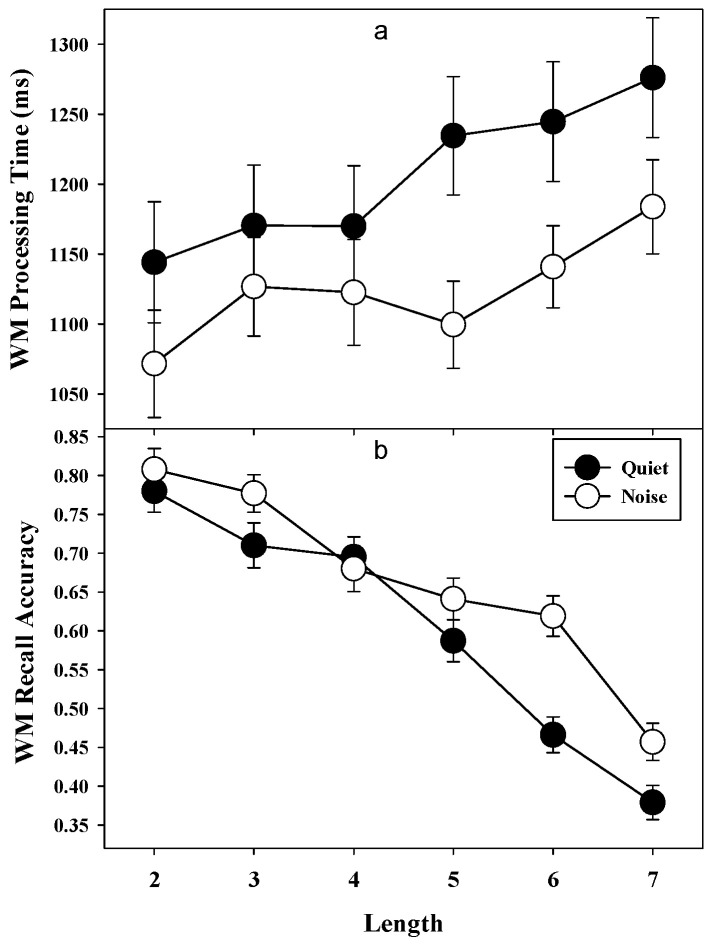
Mean processing time (panel (**a**)) and recall accuracy (panel (**b**)) for the working memory task by condition and set length. Error bars depict one standard error of mean. WM = Working memory.

*Processing time.* Response time for processing in the WM task was investigated with 2 (condition: quiet vs. noise) X 6 (block length: 2, 3, 4, 5, 6, 7) repeated measures ANOVA. There was a main effect of condition, *F*(1, 52) = 10.1, *MSE* = 1,06,709, *p* < 0.01, *η*^2^ = 0.16, indicating RT was faster in noise (M = 1134.3 ms, SD = 203.1) than in quiet condition (*M* = 1220.3 ms, *SD* = 275.5). Main effect for block length was also significant, *F*(5, 260) = 6.7, *MSE* = 28,175, *p* < 0.001, *η*^2^ = 0.11. There was a small but significant positive correlation between processing speed and list length for both quiet (r = 0.15, 95% CI (.07, 0.22), *p* < 0.01) and noise condition (r = 0.11, 95% CI (.003, 022), *p* < 0.05), suggesting that RT increased slightly with increase in list length (Figure 2a). There was no significant condition by list length interaction, *F*(5, 260) = 1.3, *MSE* = 25,195, *p* > 0.26, *η*^2^ = 0.02.

*Processing accuracy.* Accuracy of processing in WM was also subjected to 2 (condition: quiet vs. noise) X 6 (list length: 2, 3, 4, 5, 6, 7) repeated measures ANOVA. Main effect of condition was not significant, *F*(1, 52) = 2.0, *MSE* = 0.043, *p* > 0.16, *η*^2^ = 0.03, indicating that processing accuracy was not significantly different between noise (*M* = 0.86, *SD* = 0.09) and quiet condition (*M* = 0.84, *SD* = 0.11).

### 3.3. Correlation Analysis between WM and Attention Switching

Correlation analysis was performed using Pearson product moment correlation analysis. Table 2 shows the correlations among all variables for quiet and noise conditions. Looking at the relationship between recall and processing aspects of the WM task indicated a positive relation between WM recall and processing accuracy in both quiet and noise. However, there was no significant relationship between WM recall and processing speed, but processing speed was negatively related to processing accuracy. WM recall was positively related only to AS updating accuracy. Switch RT in the AS task was positively related to AS updating accuracy, but not to nonswitch RT.

### 3.4. Listening Comprehension

**Figure 3:**
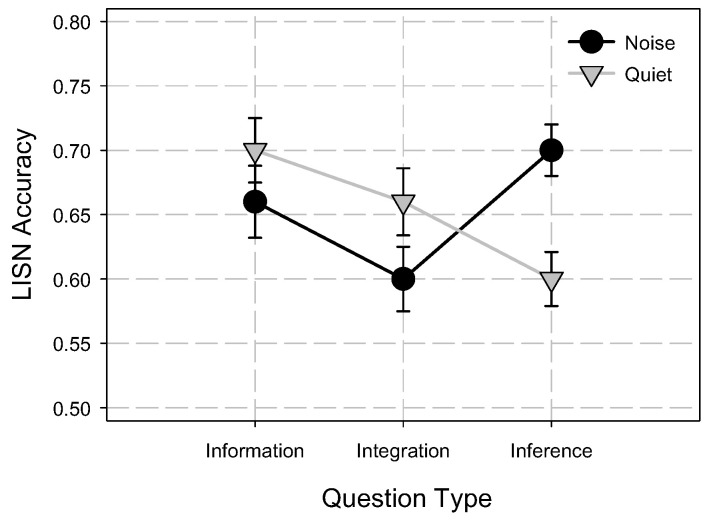
Mean accuracy for information, integration, and inference questions in the listening comprehension test. Error bars depict one standard error of mean. LISN = Lecture Interview and Spoken Narratives test.

Listening comprehension accuracy scores were subjected to 2 (condition: quiet vs. noise) X 3 (question: information. integration, inference) repeated measures ANOVA. Main effect of condition was not significant, *F*(1, 52) = 0.02, *MSE* = 0.02, *p* > 0.88, *η*^2^ = 0.00, indicating that overall accuracy was not significantly different between noise (*M* = 0.65, *SD* = 0.15) and quiet (*M* = 0.65, *SD* = 0.14) condition. However there was a significant main effect of question type, *F*(2, 104) = 6.0, *MSE* = 0.013, *p* < 0.01, *η*^2^ = 0.10. There was also a significant condition by question type interaction, *F*(2, 104) = 14.7, *MSE* = 0.013, *p* < 0.001, *η*^2^ = 0.22. Post-hoc analysis of condition by question type was examined using Tukey–Kramer multiple comparison tests. Results indicated that only accuracy for inference questions was significantly higher in noise than in quiet condition (Mean Difference = 0.10, 95% CI (0.03, 0.16)). Accuracy for information and integration questions in quiet was not significantly different from noise condition (Figure 3).

## 4. Discussion

The goal of the present study was to understand the effects of distracting noise on listening comprehension and auditory-based cognitive task performance, while controlling for speech intelligibility. The present study revealed several important and novel findings. First, adding noise increased the speed of information processing in both auditory AS and WM tasks. Second, accuracy of updating in the AS task was disrupted by the presence of noise. Third, WM capacity measured as the accuracy of recall of to-be-remembered digits was better in the presence of noise. Fourth, participants made more accurate inferences during listening in noise than in quiet. Importantly, stimulus identification accuracy in the AS task and processing accuracy in the WM task were identical in both quiet and noise conditions, indicating that the effect of noise on these cognitive tasks was not due to decreased speech intelligibility. Finally, in the presence of noise, switch RT in the AS task and processing RT in WM task were both significantly shorter than in quiet, suggesting that noise might have led to faster information processing during cognitive tasks.

### 4.1. Effect of Noise on Auditory Attention Switching and Working Memory

One finding that was apparent in both the AS and WM data was that adding fluctuating background noise significantly increased the speed of processing. This increase in speed of information processing had very different outcomes on accuracy of AS updating and WM recall. The reason for increased processing speed in the presence of noise may be attributed to the energetic/arousal theory of noise [74,75,76]. According to this theory, noise increases the arousal state of an individual [75,77]. Examples of physiological evidence of arousal in noise include increased skin resistance, pulse rate, muscle tension, etc. [78]. One of the important findings with regard to arousal and its relation to cognitive performance was the Yerkes–Dodson Law [79]. According to the Yerkes–Dodson Law, arousal and cognitive performance are related with an inverted U-shaped function and the improved performance range with increase in arousal varies with the complexity of the task. According to this, individuals’ performance is best at some arousal level and the performance falls above or below that optimal state. This optimal arousal state varies depending on the complexity of the task. Increase in arousal level also tends to be associated with reduced response criteria, which might lead to faster, but inaccurate responses [74]. According to Posner and colleagues [80,81], increased arousal/alertness does not affect the quality of decision making during a task, but it does increase the speed at which the decision is reached. Researchers [82] have studied the perception of subjects’ own response time in the presence of noise. Effect of loud noise on subjects’ choice RT and their own judgments were compared. Interestingly, subjects described their RT to be slower, whereas, actually, their RT was significantly faster in the presence of noise. Increased speed of processing or decision-making in the presence of noise reported in previous studies was consistent with the results of the present study; both switch RT in the AS task and processing RT on the WM task were quicker in noise than in quiet condition. This may be attributed to participants’ increased arousal/alertness in the presence of noise leading to faster information processing during these tasks. Furthermore, the results of the present study are consistent with the load theory of attention and cognitive control [83,84], which suggests that in the presence of an irrelevant distractor, individuals’ focus of attention depends on the information load in the relevant task. Hence, the improved performance in AS switch RT and WM accuracy found in the presence of non-informational noise may suggest increased efficiency of the attention mechanism.

The main goal of the current study was to understand the interference of fluctuating background noise on auditory-based cognitive task performance. Of specific interest were how noise affects executive functions such as AS and whether the effectiveness of AS in noise is related to recall of items in the WM task. In the AS task participants were asked to keep a running count of two categories of words presented discretely (nouns or verbs). Since attention can only be focused on one item at a time [36,85], an attention switch had to be made when updating a count from a different category. Participants had two running counts in their WM, one for each category (nouns and verbs) of words. This required them to focus their attention to the appropriate count, update that count, rehearse the other count, and finally respond to get the next stimulus [36,67]. During a switch trial, where the category of the word changed, the focus of attention had to be reoriented/switched from one count to the other, whereas in nonswitch trials the same count was updated successively, requiring no such attention reorientation/switch. Consistent with the previous findings in visual AS tasks [36,67], RT was longer during switch trials than in non-switch trials for both quiet and noise conditions. This time cost, referred to as switch cost, was an estimate of an attention switching mechanism that is thought to operate within WM [36,67,86].

It is evident from the results that irrelevant background noise did significantly reduce the switch cost. Reduced switch cost in noise indicated that participants were faster in switching their attention and updating their WM in noise than in quiet. As presented in Figure 3, an important point to note here is that the reduced switch cost in noise was mainly due to faster attention switching during switch trials, since non-switch RT was not different between the quiet and noise conditions. In other words, participants were switching their attention and updating their WM counts faster in noise whenever the word category changed. While the participants’ AS and updating were faster in noise, their accuracy of retrieving the updated counts from WM during recall was significantly poorer in noise than in quiet. Since AS updating accuracy was positively related to only switch RT, one may speculate that rapid switching of attention between the categories of words in noise compared to quiet condition might have reduced the time available to rehearse the counts in WM leading to reduced updating accuracy in AS task.

Similar to the AS task, processing RT in the WM task was faster in the presence of fluctuating noise. Considering that the duration of the stimulus presentation and inter-stimulus interval between the stimuli (digits and words) was constant for both quiet and noise, these results clearly indicate that participants were processing the words faster in noise. Despite faster RT, processing accuracy in the WM task was not different in both conditions. These findings should come as no surprise because the sentence structures were well within the linguistic grasp of the participants. Interestingly, participants were able to serially recall more digits in the presence of fluctuating noise than in quiet. To understand this effect, let us look at the WM task closely. In the auditory WM task, participants were asked to actively maintain digits along with concurrent processing of words presented sequentially to select one of the three choices. At the end of each block, they were asked to recall the digits in the same order. The WM task requires attention switching to successfully perform both processing of words and maintenance of digits [27,35,41]. Since attention resources are limited and cannot be focused on both processing and maintenance at the same time, they need to be shared between maintenance and processing [22,55,67,68,87]. When the focus of attention was switched away from maintenance to processing during the WM task, stored digits in WM were likely subject to decay [35,43,45]. Maintaining digits for future recall required participants to frequently switch their attention away from processing to refresh items in WM. This meant that the more time the processing component of the WM task captured attention, the less time was available for maintenance or rehearsal of digits, leading to poorer recall performance [33]. This was especially true when the total time allowed for both processing and maintenance were fixed as in the present study. Several models of WM have highlighted the importance of attention control between items that are within the focus and those that are currently active but outside the focus of attention [35,40,43,45,67,68]. Consistent with prior research [36,67,88], we also found that accuracy of AS updating was positively related with WM recall accuracy in both quiet and noise conditions. Even though switch RT was not related to WM recall accuracy, the partial correlation between switch RT and WM recall accuracy was significant in quiet (*r* = −0.51, *p* < 0.01) and noise (*r* = −0.37, *p* < 0.01) conditions when controlling for AS updating accuracy. This suggests that when updating accuracy was equated, individuals with faster attention switching ability recalled more digits in WM task.

One possible explanation for better WM recall of digits in noise may be due to faster attention switching in noise during the processing part of WM task. As accuracy of processing information during the WM task was not affected by noise, but the speed of processing was significantly faster, participants might have processed the words presented in series faster and used the time between the presentations of word for rehearsing digits. This rapid attention switching from processing to refreshing items in the memory during the processing part (gaps between the words presentation) of the WM task would have prevented loss of information in WM. These results may indicate that individual differences in speed of switching attention to and updating information outside the focus are related to differences in WM capacity.

### 4.2. Effect of Noise on Listening Comprehension

Listening comprehension was measured using extended spoken passages in quiet and in the presence of fluctuating ICRA noise. Participants’ comprehension was measured for information, integration, and inference question types. The results of the present study in quiet were consistent with the original published LISN test data [72]. Listeners performed best on information questions followed by integration questions and poorest on inference questions in quiet condition. However, the most intriguing finding of the present study was in the noise condition. Contrary to listening comprehension performance in quiet, listeners’ performance in the presence of noise was best on inference questions than on information or integration questions. Listeners’ performance for inference questions was significantly better in noise than in quiet condition (Figure 3). Several studies have shown that making inferences during reading and listening relies heavily on WM [89,90,91,92]. Researchers [93] who have explored the relationship between WM capacity and reading comprehension in 206 participants have argued that, the reason WM capacity predicted complex cognitive activities, such as reading comprehension, was because the storage and processing capacity of WM is important for retaining new information, making inferences, and integrating new information with the knowledge from LTM. In the present study, participants’ recall on both WM tasks were significantly better in noise than in quiet, which suggested that participants might have been able to utilize the increased WM capacity to their advantage in answering the inference questions in noise. That is, improved performance for inference questions in noise may be attributed to increased/better WM deployment in the presence of noise.

## 5. Conclusions

The goal of this research study was to understand the effect of noise on cognitive and listening comprehension performance. The level of noise was individually adjusted to ensure that the speech intelligibility was at least 90%. The results revealed some novel findings. In the presence of noise, speed of information processing was significantly faster for both AS and WM tasks. WM capacity and inference making ability were better, but AS updating accuracy was affected in the presence of noise. The pattern of these results was consistent with the notion that noise increased the arousal level of individuals, which in turn led to faster processing in the WM task and rapid shifting of attention in the AS task; while AS and updating were faster in noise, the accuracy of retrieving the updated counts was significantly poorer in noise which suggested that that speeded AS caused more errors in updating the items in WM during the AS task. Also in the WM task, participants who performed the processing part more accurately used less time for processing and more effectively switched their attention to rehearsing the items in WM resulting in better recall accuracy in noise. The results obtained in this study apply to non-informational background noise, and additional research is required to examine these findings using other types of background noise that can cause informational masking.

## Figures and Tables

**Table 1:** Descriptive data for all the experimental tasks for both quiet and noise condition.

Measure	Condition	M (SD)	Range	Skew	Kurtosis
Attention Switching					
Updating Acc	Q	0.76 (0.20)	[0.11, 1]	−1.3	1.5
	N	0.68 (0.21)	[0.16, 0.96]	−0.72	−0.31
Switch RT (ms)	Q	1141.9 (224.1)	[683.7, 1579.1]	−0.06	−0.99
	N	1084.3 (171.7)	[701.1, 1422.4]	−0.16	−0.65
Nonswitch RT (ms)	Q	1007.0 (180.4)	[701.1, 1497.1]	0.32	−0.48
	N	997.8 (142.4)	[699.8, 1319.3]	−0.02	−0.76
Working Memory					
Recall Acc	Q	0.68 (0.16)	[0.19, 0.95]	−0.54	0.26
	N	0.73 (0.15)	[0.38, 0.97]	−0.49	−0.63
Processing Acc	Q	0.84 (0.11)	[0.52, 1.0]	−1.0	0.95
	N	0.86 (0.09)	[0.56, 0.99]	−0.96	0.65
Processing RT (ms)	Q	1220.3 (275.5)	[541.0, 1676.1]	−0.35	−0.64
	N	1134.3 (203.1)	[694.8, 1524.5]	−0.02	−0.55
Listening Comprehension					
Information Acc	Q	0.70 (0.18)	[0.33, 1]	0.00	−0.87
	N	0.66 (0.21)	[0.08, 1]	−0.58	−0.08
Integration Acc	Q	0.66 (0.19)	[0.17, 1]	−0.14	−0.52
	N	0.60 (0.18)	[0.25, 1]	0.28	−0.17
Inference Acc	Q	0.60 (0.15)	[0.33, 0.92]	−0.16	−0.58
	N	0.70 (0.14)	[0.25, 0.92]	−0.67	0.75
Overall Acc	Q	0.65 (0.15)	[0.33, 0.94]	−0.32	−0.76
	N	0.65 (0.15)	[0.25, 0.92]	−0.25	−0.45

Note: Acc = Accuracy; RT = Reaction time; Q = Quiet; N = Noise.

**Table 2:** Correlation among measures in quiet (below the diagonal) and noise (above the diagonal).

	Variables	1	2	3	4	5	6
1	WM Recall	1	0.76 **	−0.24	0.44 **	−0.21	−0.08
2	WM RT	0.66 **	1	−0.43 **	0.29 *	−0.34 *	−0.27 *
3	WM Acc	−0.26	−0.53 **	1	0.02	0.54 **	0.35 **
4	AS Updating	0.47 **	0.35 **	0.02	1	0.24	0.44 **
5	AS Sw RT	−0.20	−0.39 **	0.48 **	0.41 **	1	0.88 **
6	AS Nsw RT	−0.27 *	−0.50 **	0.61 **	0.23	0.87 **	1

* *p* < 0.01, ** *p* < 0.001, *N* = 53. WM = Working memory; Acc = Accuracy; RT = Reaction time; AS = Attention Switching; Sw = Switch; Nsw = Nonswitch.

## Data Availability

The data presented in this study are available on request from the corresponding author. The data will be made publicly available after publication at https://library.usu.edu/data-management/index.
